# Supplementary material for: Hot spot prediction in protein-protein interactions by an ensemble system
Source: BMC Syst Biol. 2018 Dec 31;12(Suppl 9):132. doi: 10.1186/s12918-018-0665-8 (PMC6311905; doi:10.1186/s12918-018-0665-8)
Supplement: Supplementary file 2 — All relevant MATLAB code. A simple Matlab implement of our predictor. (ZIP 19,742 kb) [file 12918_2018_665_MOESM2_ESM.zip › Additional file 2/Read ASA/ASA website/dbMPIKT/1KN0A.html]

 NetSurfP - Protein Surface Accessibility and Secondary Structure Predictions

  

|  |  |  |
| --- | --- | --- |
|  |  | NetSurfP - Protein Surface Accessibility and Secondary Structure Predictions  Technical University of Denmark |

  


---

```
# For publication of results, please cite:
# A generic method for assignment of reliability scores applied to solvent accessibility predictions.
# Bent Petersen, Thomas Nordahl Petersen, Pernille Andersen, Morten Nielsen and Claus Lundegaard
# BMC Structural Biology 2009, 9:51 doi:10.1186/1472-6807-9-51
#
# Column 1: Class assignment - B for buried or E for Exposed - Threshold: 25% exposure, but not based on RSA
# Column 2: Amino acid
# Column 3: Sequence name
# Column 4: Amino acid number
# Column 5: Relative Surface Accessibility - RSA
# Column 6: Absolute Surface Accessibility
# Column 7: Z-fit score for RSA prediction
# Column 8: Probability for Alpha-Helix
# Column 9: Probability for Beta-strand
# Column 10: Probability for Coil
E M  1KN0A                  1    0.782 156.458  -1.568   0.003   0.003   0.994
E S  1KN0A                  2    0.561  65.784  -1.269   0.018   0.019   0.964
E G  1KN0A                  3    0.521  41.034  -1.495   0.115   0.016   0.868
E T  1KN0A                  4    0.417  57.880  -1.333   0.268   0.043   0.689
E E  1KN0A                  5    0.553  96.522  -0.782   0.502   0.102   0.396
E E  1KN0A                  6    0.492  85.952  -0.998   0.502   0.102   0.396
B A  1KN0A                  7    0.213  23.417  -1.079   0.428   0.171   0.402
B I  1KN0A                  8    0.267  49.339  -0.838   0.428   0.171   0.402
E L  1KN0A                  9    0.388  71.079  -1.148   0.428   0.171   0.402
E G  1KN0A                 10    0.474  37.320  -0.797   0.278   0.093   0.628
E G  1KN0A                 11    0.375  29.489  -1.977   0.191   0.086   0.723
E R  1KN0A                 12    0.349  79.875  -0.874   0.191   0.086   0.723
E D  1KN0A                 13    0.466  67.194  -1.023   0.199   0.152   0.649
E S  1KN0A                 14    0.356  41.676  -1.473   0.113   0.087   0.800
B H  1KN0A                 15    0.181  32.978   0.201   0.113   0.087   0.800
E P  1KN0A                 16    0.379  53.794  -0.217   0.113   0.087   0.800
E A  1KN0A                 17    0.329  36.278  -1.419   0.113   0.087   0.800
E A  1KN0A                 18    0.390  42.967  -0.753   0.053   0.043   0.903
E G  1KN0A                 19    0.506  39.854  -0.886   0.053   0.043   0.903
E G  1KN0A                 20    0.691  54.382  -1.729   0.018   0.019   0.964
E G  1KN0A                 21    0.342  26.876  -1.651   0.018   0.019   0.964
E S  1KN0A                 22    0.576  67.519  -1.464   0.018   0.047   0.935
B V  1KN0A                 23    0.247  37.964  -0.879   0.018   0.047   0.935
E L  1KN0A                 24    0.323  59.160  -0.185   0.018   0.047   0.935
E C  1KN0A                 25    0.291  40.913  -1.853   0.018   0.088   0.893
B F  1KN0A                 26    0.214  43.010  -1.234   0.018   0.088   0.893
B G  1KN0A                 27    0.263  20.667  -1.452   0.052   0.084   0.864
E Q  1KN0A                 28    0.377  67.368  -0.492   0.113   0.087   0.800
B C  1KN0A                 29    0.247  34.693   0.118   0.184   0.043   0.773
E Q  1KN0A                 30    0.553  98.802   0.485   0.354   0.048   0.598
B Y  1KN0A                 31    0.159  33.893   0.286   0.354   0.048   0.598
B T  1KN0A                 32    0.197  27.268   0.500   0.430   0.016   0.555
E A  1KN0A                 33    0.397  43.771   0.010   0.923   0.002   0.076
E E  1KN0A                 34    0.640 111.860   1.003   0.970   0.001   0.030
E E  1KN0A                 35    0.298  52.113   0.752   0.970   0.001   0.030
B Y  1KN0A                 36    0.145  31.072   0.757   0.970   0.001   0.030
E Q  1KN0A                 37    0.469  83.763   0.491   0.970   0.001   0.030
E A  1KN0A                 38    0.423  46.637   1.717   0.970   0.001   0.030
B I  1KN0A                 39    0.046   8.510   0.468   0.970   0.001   0.030
B Q  1KN0A                 40    0.226  40.346   0.614   0.970   0.001   0.030
E K  1KN0A                 41    0.492 101.184   0.856   0.970   0.001   0.030
E A  1KN0A                 42    0.299  32.950   1.539   0.970   0.001   0.030
B L  1KN0A                 43    0.051   9.356   0.192   0.923   0.002   0.076
E R  1KN0A                 44    0.497 113.744  -0.124   0.858   0.002   0.139
E Q  1KN0A                 45    0.571 102.034   0.179   0.694   0.003   0.303
E R  1KN0A                 46    0.448 102.684  -0.639   0.502   0.002   0.495
B L  1KN0A                 47    0.221  40.483  -0.519   0.115   0.016   0.868
E G  1KN0A                 48    0.366  28.773  -0.562   0.018   0.019   0.964
E P  1KN0A                 49    0.449  63.727  -1.112   0.018   0.047   0.935
E E  1KN0A                 50    0.511  89.219  -1.570   0.021   0.279   0.699
E Y  1KN0A                 51    0.309  66.012  -0.406   0.022   0.359   0.619
B I  1KN0A                 52    0.116  21.442  -0.453   0.021   0.451   0.528
E S  1KN0A                 53    0.325  38.078  -0.790   0.021   0.451   0.528
E S  1KN0A                 54    0.402  47.150  -0.897   0.022   0.552   0.426
B R  1KN0A                 55    0.290  66.341   0.045   0.022   0.552   0.426
E M  1KN0A                 56    0.466  93.267  -1.434   0.021   0.451   0.528
E A  1KN0A                 57    0.360  39.650  -1.292   0.019   0.141   0.840
E G  1KN0A                 58    0.635  49.990  -1.894   0.018   0.047   0.935
E G  1KN0A                 59    0.585  46.047  -2.569   0.018   0.019   0.964
E G  1KN0A                 60    0.533  41.939  -2.318   0.018   0.088   0.893
B Q  1KN0A                 61    0.308  55.062  -1.415   0.021   0.279   0.699
E K  1KN0A                 62    0.380  78.207   0.236   0.023   0.655   0.322
B V  1KN0A                 63    0.141  21.656   0.304   0.021   0.756   0.223
B C  1KN0A                 64    0.149  20.892   0.005   0.021   0.756   0.223
B Y  1KN0A                 65    0.152  32.418  -0.488   0.023   0.655   0.322
B I  1KN0A                 66    0.077  14.226   0.099   0.074   0.484   0.442
E E  1KN0A                 67    0.307  53.668  -0.241   0.118   0.150   0.732
B G  1KN0A                 68    0.081   6.335  -2.233   0.622   0.015   0.363
E H  1KN0A                 69    0.298  54.133  -0.323   0.802   0.014   0.185
E R  1KN0A                 70    0.250  57.136   1.280   0.879   0.010   0.111
B V  1KN0A                 71    0.026   3.950   0.646   0.950   0.028   0.022
B I  1KN0A                 72    0.043   8.029   0.747   0.950   0.028   0.022
E N  1KN0A                 73    0.327  47.931   0.534   0.938   0.007   0.055
B L  1KN0A                 74    0.094  17.211   0.741   0.879   0.010   0.111
B A  1KN0A                 75    0.021   2.347   0.450   0.858   0.002   0.139
B N  1KN0A                 76    0.111  16.236  -0.331   0.858   0.002   0.139
E E  1KN0A                 77    0.344  60.062   0.378   0.858   0.002   0.139
B M  1KN0A                 78    0.047   9.425   0.321   0.879   0.010   0.111
B F  1KN0A                 79    0.062  12.463   0.669   0.717   0.014   0.269
E G  1KN0A                 80    0.297  23.366  -1.007   0.268   0.043   0.689
B Y  1KN0A                 81    0.199  42.484  -0.846   0.113   0.043   0.844
E N  1KN0A                 82    0.396  58.004  -1.083   0.018   0.019   0.964
B G  1KN0A                 83    0.253  19.950  -1.832   0.018   0.047   0.935
B W  1KN0A                 84    0.125  30.087  -0.493   0.056   0.142   0.802
B A  1KN0A                 85    0.167  18.425  -1.109   0.022   0.359   0.619
B H  1KN0A                 86    0.186  33.833  -0.390   0.021   0.451   0.528
E S  1KN0A                 87    0.357  41.899   0.638   0.021   0.756   0.223
B I  1KN0A                 88    0.100  18.426   0.206   0.018   0.846   0.136
E T  1KN0A                 89    0.292  40.473   0.612   0.011   0.918   0.071
E Q  1KN0A                 90    0.533  95.212  -0.042   0.001   0.900   0.099
B Q  1KN0A                 91    0.187  33.380   0.925   0.002   0.816   0.182
E N  1KN0A                 92    0.477  69.833  -0.443   0.003   0.718   0.279
B V  1KN0A                 93    0.187  28.788   1.025   0.004   0.616   0.381
E D  1KN0A                 94    0.385  55.507  -0.457   0.023   0.655   0.322
B F  1KN0A                 95    0.227  45.519  -0.268   0.023   0.655   0.322
E V  1KN0A                 96    0.355  54.579  -0.253   0.018   0.846   0.136
B D  1KN0A                 97    0.234  33.662  -0.446   0.018   0.846   0.136
E L  1KN0A                 98    0.398  72.874  -0.303   0.023   0.655   0.322
E N  1KN0A                 99    0.656  96.097  -0.139   0.005   0.262   0.733
E N  1KN0A                100    0.688 100.665  -0.050   0.004   0.085   0.910
E G  1KN0A                101    0.438  34.494  -0.634   0.004   0.197   0.799
E K  1KN0A                102    0.411  84.563   1.080   0.002   0.816   0.182
B F  1KN0A                103    0.107  21.415   0.634   0.001   0.900   0.099
B Y  1KN0A                104    0.172  36.778  -0.335   0.001   0.959   0.040
B V  1KN0A                105    0.030   4.596   0.212   0.001   0.959   0.040
B G  1KN0A                106    0.062   4.911   0.249   0.001   0.959   0.040
B V  1KN0A                107    0.035   5.333   0.103   0.001   0.959   0.040
B C  1KN0A                108    0.062   8.691   0.246   0.001   0.959   0.040
B A  1KN0A                109    0.015   1.708   1.187   0.001   0.959   0.040
B F  1KN0A                110    0.108  21.656   0.834   0.001   0.959   0.040
B V  1KN0A                111    0.024   3.735   0.759   0.001   0.959   0.040
B R  1KN0A                112    0.195  44.586   1.347   0.001   0.959   0.040
B V  1KN0A                113    0.044   6.701   0.558   0.001   0.959   0.040
B Q  1KN0A                114    0.188  33.613   1.081   0.001   0.959   0.040
B L  1KN0A                115    0.055  10.052   0.777   0.001   0.900   0.099
E K  1KN0A                116    0.462  95.054   0.978   0.004   0.420   0.576
E D  1KN0A                117    0.478  68.822  -1.088   0.004   0.085   0.910
E G  1KN0A                118    0.438  34.471  -1.608   0.018   0.047   0.935
E S  1KN0A                119    0.377  44.138  -1.092   0.021   0.279   0.699
E Y  1KN0A                120    0.374  79.924  -0.103   0.023   0.655   0.322
E H  1KN0A                121    0.374  67.994  -0.036   0.021   0.756   0.223
E E  1KN0A                122    0.379  66.229  -0.398   0.022   0.552   0.426
E D  1KN0A                123    0.383  55.176  -1.951   0.021   0.451   0.528
B V  1KN0A                124    0.210  32.216  -0.364   0.022   0.359   0.619
B G  1KN0A                125    0.121   9.491  -2.774   0.021   0.451   0.528
B Y  1KN0A                126    0.174  37.098  -0.890   0.074   0.484   0.442
B G  1KN0A                127    0.140  11.042  -1.556   0.074   0.484   0.442
E V  1KN0A                128    0.368  56.546   0.018   0.074   0.484   0.442
B S  1KN0A                129    0.211  24.717  -0.721   0.066   0.296   0.638
E E  1KN0A                130    0.482  84.240  -0.507   0.064   0.216   0.721
E G  1KN0A                131    0.449  35.297  -0.836   0.018   0.088   0.893
B L  1KN0A                132    0.111  20.397   0.020   0.339   0.016   0.645
E K  1KN0A                133    0.514 105.668   0.227   0.502   0.002   0.495
E S  1KN0A                134    0.421  49.388  -0.381   0.600   0.003   0.397
B K  1KN0A                135    0.187  38.507   0.044   0.858   0.002   0.139
B A  1KN0A                136    0.166  18.337  -1.289   0.923   0.002   0.076
E L  1KN0A                137    0.418  76.572   0.805   0.923   0.002   0.076
B S  1KN0A                138    0.073   8.591  -0.919   0.970   0.001   0.030
B L  1KN0A                139    0.102  18.621   0.658   0.923   0.002   0.076
E E  1KN0A                140    0.389  67.941   0.335   0.923   0.002   0.076
E K  1KN0A                141    0.455  93.635   0.878   0.858   0.002   0.139
B A  1KN0A                142    0.051   5.598  -0.143   0.858   0.002   0.139
E R  1KN0A                143    0.277  63.479   0.992   0.858   0.002   0.139
E K  1KN0A                144    0.359  73.764   0.751   0.879   0.010   0.111
E E  1KN0A                145    0.369  64.517   0.865   0.802   0.014   0.185
B A  1KN0A                146    0.084   9.246  -0.183   0.717   0.014   0.269
B V  1KN0A                147    0.096  14.694   0.464   0.717   0.014   0.269
B T  1KN0A                148    0.213  29.474  -0.111   0.622   0.015   0.363
E D  1KN0A                149    0.476  68.534   0.295   0.858   0.002   0.139
B G  1KN0A                150    0.062   4.903  -0.947   0.970   0.001   0.030
B L  1KN0A                151    0.063  11.462   0.203   0.970   0.001   0.030
E K  1KN0A                152    0.275  56.485   1.241   0.970   0.001   0.030
E R  1KN0A                153    0.326  74.562   1.003   0.970   0.001   0.030
B A  1KN0A                154    0.024   2.689   0.391   0.970   0.001   0.030
B L  1KN0A                155    0.049   8.972  -1.116   0.923   0.002   0.076
E R  1KN0A                156    0.310  70.990   0.146   0.923   0.002   0.076
B S  1KN0A                157    0.140  16.431  -0.699   0.782   0.003   0.216
B F  1KN0A                158    0.052  10.376  -0.376   0.600   0.003   0.397
B G  1KN0A                159    0.150  11.781  -1.588   0.406   0.004   0.590
B N  1KN0A                160    0.230  33.613  -1.587   0.502   0.002   0.495
B A  1KN0A                161    0.152  16.706  -1.515   0.522   0.016   0.462
B L  1KN0A                162    0.082  14.978  -0.896   0.430   0.016   0.555
B G  1KN0A                163    0.122   9.625  -2.034   0.339   0.016   0.645
B N  1KN0A                164    0.166  24.244  -1.301   0.339   0.016   0.645
B C  1KN0A                165    0.060   8.382  -0.082   0.455   0.046   0.498
B I  1KN0A                166    0.095  17.538  -0.374   0.561   0.047   0.393
B L  1KN0A                167    0.207  37.938   0.142   0.455   0.046   0.498
B D  1KN0A                168    0.242  34.872  -0.050   0.561   0.047   0.393
E K  1KN0A                169    0.328  67.428   0.513   0.879   0.010   0.111
E D  1KN0A                170    0.551  79.385   0.344   0.923   0.002   0.076
B Y  1KN0A                171    0.141  30.068   0.751   0.923   0.002   0.076
B L  1KN0A                172    0.105  19.226   0.185   0.970   0.001   0.030
E R  1KN0A                173    0.513 117.500   0.915   0.923   0.002   0.076
E S  1KN0A                174    0.501  58.729   1.086   0.923   0.002   0.076
B L  1KN0A                175    0.080  14.685   0.247   0.858   0.002   0.139
E N  1KN0A                176    0.376  55.061  -0.342   0.782   0.003   0.216
E K  1KN0A                177    0.545 112.127   0.657   0.694   0.003   0.303
B L  1KN0A                178    0.248  45.445   0.002   0.622   0.015   0.363
E P  1KN0A                179    0.321  45.493  -0.715   0.339   0.016   0.645
E R  1KN0A                180    0.617 141.362  -0.107   0.181   0.016   0.803
E Q  1KN0A                181    0.443  79.156  -0.212   0.115   0.016   0.868
E L  1KN0A                182    0.487  89.096  -0.533   0.018   0.019   0.964
E P  1KN0A                183    0.330  46.784  -0.410   0.018   0.019   0.964
E L  1KN0A                184    0.371  67.875  -0.271   0.018   0.019   0.964
E E  1KN0A                185    0.669 116.927   1.142   0.053   0.043   0.903
B V  1KN0A                186    0.127  19.535   0.628   0.113   0.043   0.844
E D  1KN0A                187    0.471  67.929   0.600   0.058   0.017   0.925
E L  1KN0A                188    0.474  86.863  -0.445   0.339   0.016   0.645
E T  1KN0A                189    0.607  84.163  -1.047   0.339   0.016   0.645
E K  1KN0A                190    0.430  88.472   0.631   0.430   0.016   0.555
B A  1KN0A                191    0.119  13.136  -0.438   0.455   0.046   0.498
E K  1KN0A                192    0.318  65.351   0.247   0.278   0.093   0.628
E R  1KN0A                193    0.372  85.119   0.145   0.191   0.086   0.723
E Q  1KN0A                194    0.422  75.405   0.613   0.053   0.043   0.903
E D  1KN0A                195    0.407  58.577  -0.789   0.115   0.016   0.868
E L  1KN0A                196    0.381  69.798  -1.210   0.181   0.016   0.803
E E  1KN0A                197    0.486  84.887  -0.698   0.339   0.016   0.645
E P  1KN0A                198    0.439  62.365  -0.633   0.430   0.016   0.555
E S  1KN0A                199    0.384  44.970  -0.102   0.455   0.046   0.498
E V  1KN0A                200    0.432  66.445   0.208   0.455   0.046   0.498
E E  1KN0A                201    0.447  78.161  -0.127   0.561   0.047   0.393
E E  1KN0A                202    0.493  86.145   0.054   0.522   0.016   0.462
E A  1KN0A                203    0.297  32.685  -1.350   0.522   0.016   0.462
E R  1KN0A                204    0.443 101.539  -1.028   0.430   0.016   0.555
E Y  1KN0A                205    0.466  99.648  -0.410   0.257   0.016   0.727
E N  1KN0A                206    0.517  75.630  -0.457   0.109   0.005   0.886
E S  1KN0A                207    0.549  64.355  -0.676   0.109   0.005   0.886
B C  1KN0A                208    0.201  28.234  -1.311   0.109   0.005   0.886
E R  1KN0A                209    0.491 112.508   0.248   0.058   0.017   0.925
E P  1KN0A                210    0.668  94.818  -0.725   0.058   0.017   0.925
E N  1KN0A                211    0.667  97.678  -1.861   0.053   0.005   0.942
E M  1KN0A                212    0.681 136.368  -1.662   0.003   0.003   0.994

Explain the output.  Go back.
```

---
